# Comprehensive value assessment of drugs using a multi-criteria decision analysis: An example of targeted therapies for metastatic colorectal cancer treatment

**DOI:** 10.1371/journal.pone.0225938

**Published:** 2019-12-12

**Authors:** Jason C. Hsu, Jia-Yu Lin, Peng-Chan Lin, Yang-Cheng Lee

**Affiliations:** 1 School of Pharmacy and Institute of Clinical Pharmacy and Pharmaceutical Sciences, College of Medicine, National Cheng Kung University, Tainan, Taiwan; 2 Department of Pharmacy, National Cheng Kung University Hospital, Tainan, Taiwan; 3 Department of Internal Medicine, National Cheng Kung University Hospital, Tainan, Taiwan; 4 Department of Internal Medicine, Tainan Municipal Hospital, Tainan, Taiwan; Chang Gung Memorial Hospital at Linkou, TAIWAN

## Abstract

**Objective:**

This study is aimed toward establishing a decision-making model with multiple criteria for appraisal and reimbursement to compare the attitudes of different stakeholders toward various dimensions and criteria and to evaluate the five targeted therapies (bevacizumab, cetuximab, panitumumab, aflibercept, and regorafenib) for metastatic colorectal cancer.

**Method:**

This study is a multi-criteria decision analysis (MCDA) using a model that includes three dimensions and nine criteria. Both the overall and individual scores of the respective targeted therapies in different dimensions and criteria were calculated. A sensitivity analysis was carried out in order to evaluate the robustness of the research results. An interview-based questionnaire survey was applied to obtain the performance information for the targeted therapies and the weights of the dimensions and criteria.

**Results:**

Overall, the clinical dimension had the highest weight, followed by the economic dimension, and finally, the social dimension. In the clinical dimension, the “comparative efficacy” criterion had the highest weight; in the economic dimension, the “cost-effectiveness” criterion” was given the greatest importance; in the social dimension, the “social concern and patient needs” criterion was given more emphasis. The overall values ranked from high to low as follows: cetuximab (overall score 3.3666), bevacizumab (3.3043), panitumumab (3.2030), aflibercept (2.8923) and regorafenib (2.8366).

**Conclusions:**

A comprehensive value assessment system combining “multi-dimensional criteria,” “multi-perspectives,” and an “integrative assessment” is necessary to evaluate the value of medicines. The results showed not only the order of weights of different dimensions or criteria, but also the rankings of the value of the targeted therapies.

## Introduction

A value assessment of medicines can serve as a basis for national health insurance (NHI) payments, hospital drug procurement, clinical treatment selection, drug research, and development on the part of pharmaceutical companies. Before determining whether a drug is formally listed in the NHI, medical professionals, patients, insurance payers, drug manufacturers, and other relevant group preferences should be taken into consideration. In addition, drug-related clinical (e.g. comparative efficacy), economic (e.g. cost-effectiveness and budget impact), and social (e.g. disease treatment using existing drugs failing to meet needs) dimensions, as well as other multi-dimensional evidence are used to carry out value assessments that support decision making.[1, 2] Hospitals with a limited procurement budget should conduct drug value assessments with clear assessment criteria and transparent procurement processes. In addition, drugs with the highest level of demand and most cost-effectiveness should be chosen among many drug options.[3–5] During clinical diagnosis and treatment, a value assessment process jointly decided by the doctor and the patient should be adopted to help select the most suitable drugs.[1, 6] Furthermore, drug efficacy, quality, and cost value assessments should be conducted during the new drug development process in order to assist pharmaceutical companies with determining the most suitable research targets and methods and also assist with the adoption of corresponding R&D strategies.[7]

A multi-criteria decision analysis (MCDA) is a quantitative, structured assessment method that takes into consideration multiple criteria, including multiple view-points, in which an integrated pattern is adopted to carry out analyses [8] applicable for drug value assessments. MCDAs has been applied in many research fields, including public health, medical health-related policy formulations, drug formulary listing, value assessment, etc. [9, 10] The first paper on MCDA in the medical field literature was published in 1990. At the time, it was intended to assist in setting up related screening strategies. The consideration criteria included screening expenditures, execution results, and other qualitative criteria (e.g. execution feasibility, ethical acceptance, instantaneous information tracking, and influence arising from health education, etc.). [11] From 1994 to 1999, MCDA tool was often used in clinical assessments, such as the value assessment of the use of Isoniazid on those tested positive in the skin tuberculosis test to prevent tuberculosis [12], intermittent claudication drug treatment options [13], and other applications.

In 2001, it was applied to public health issues, and a set of control measures was set up for acquired immunodeficiency syndrome. [14] It was not until 2011 that the applications extended to drug policy formulation. At that time, it was used to help low-income countries determine which drugs should be included in the formulary listing. It turned out that the drug types recommended for inclusion in the formulary listing included anti-malarial medication, asthma medication, and antibiotics used to treat urinary tract infections. [15] In addition, the German Health Technology Assessment Institution (HTAI) integrated the opinions of patients and medical personnel and used the analytic hierarchy process (AHP) to collect the prevalence of specific medical staff and patient situations and their perspective and degree of focus on such situations. For example, medical personnel focus on preventing disease recurrence, while patients focus on how long it takes for a medication to take effect. [16]

In 2012, an academic group in Canada applied a multivariate decision-making analysis on medical and technological assessments and designed a drug assessment framework, called EVIDEM (Evidence and Value: Impact on DEcision Making). The assessment framework comprised 15 criteria (e.g. degree of disease severity, cost-effectiveness, etc.). The respective criteria were targeted and given scores on a scale of 0–3. [17] Later, Thailand’s HTAI referred to the EVIDEM assessment framework and gave different measurement descriptions targeting respective scores. Finally, it was applied as a consideration for evaluating public health plan (including drugs) execution. [18] Hence, MCDA has recently undergone rapid development, extending from clinical applications to decision-making and has been used to carry out in-depth applications and explorations.

In recent years, cancer targeted therapies have been researched, developed, launched, and covered by insurance in different countries, but this has resulted in a huge financial burden on insurance entities. However, studies that use MCDAs to evaluate the value of cancer targeted therapies still remain scarce. A number of targeted therapies have become available for the treatment of colorectal cancer. In this study, an MCDA was used to evaluate the value of existing targeted therapies for metastatic colorectal cancer. Academically, this study is a sample of an MCDA value assessment of drugs; clinically, in addition to providing targeted therapy-related uses, insight was gained into the therapeutic demand for targeted therapies that can lead to more accurate drug selection suggestions; on the policy side, insight was gained into the measurement focus of respondents with different attributes and perspectives when giving consideration to the inclusion of drugs obtaining NHI coverage, thereby serving as a decision reference for policy formulators.

According to the clinical treatment guideline released by the National Comprehensive Cancer Network, NCCN) in 2017[19], five drugs for metastatic colorectal cancer have currently been approved by the Taiwan FDA, including bevacizumab, cetuximab, panitumumab, aflibercept, and regorafenib. The therapeutic mechanisms of these drugs can be divided into three categories: (1) vascular endothelial growth factor (VEGF) inhibitors, (2) epidermal growth factor receptor (EGFR) inhibitors, and (3) multikinase inhibitors. Bevacizumab and aflibercept both fall into the vascular endothelial growth factor (VEGF) inhibitor category since they combine with the VEGF released by tumor cells in order to reduce angiogenesis; cetuximab and panitumumab fall into the epidermal growth factor receptor (EGFR) inhibitor category, since they inhibit tumor cell growth, division, and transfer. Treatment effectiveness is reduced as a result of RAS genetic mutations. Regorafenib can act on multiple kinase receptors and is the only oral drug used to treat metastatic colorectal cancer. [20–24]

## Methods

Considering the international MCDA guidelines developed by the International Society for Pharmacoeconomics and Outcomes Research (ISPOR) [25], this study included the following steps: (1) defining the decision problem, (2) selecting and structuring the related criteria, (3) weighting criteria, (4) measurement of performance, (5) scoring alternatives, (6) calculating aggregate scores, (7) dealing with uncertainty, and (8) interpretation and reporting of the results. The detailed methods by step are as follows:

### 1. Defining the decision problem

First, it is necessary to define the decision problems, content, objectives, suitable interview subjects, and the assessed drugs. This study was an attempt to gain an insight into the opinions of various stakeholders on the value assessment criteria. The existing drugs for metastatic colorectal cancer were used as targets for the purpose of constructing a value assessment mode. The interview subjects included attendees at expert consultation meetings and discussion meetings, as well as clinical experts familiar with the drugs used to treat metastatic colorectal cancer. The Taiwan FDA has approved five targeted therapies, including bevacizumab, cetuximab, panitumumab, aflibercept, and regorafenib, as assessed drugs. Clinically, bevacizumab, cetuximab, and panitumumab are mainly for first-line use; aflibercept is for second line use, and regorafenib is the for last line use. [19]

### 2. Selection and structuring criteria

When constructing assessment dimensions and criteria, in principle, comprehensive considerations are given, and repetition is avoided to achieve independence among selected criteria. Additionally, a hierarchical structure is used to present a value tree. [25] A tree diagram clearly presents all the considerations and choices as well as the final assessed drugs in the form of a hierarchical structure. Furthermore, the analytical hierarchy process, AHP, proposed by Saaty in 1971, can be presented in a decision hierarchical diagram, and it can be used to help with calculating the weights of the criteria. [26]

In the current study, first, the decision problem imagery was organized into a decision hierarchy diagram, of which the highest hierarchy was the decision objective, followed by the second hierarchy representing the assessment dimensions, the second to the last hierarchy comprising specific and quantifiable assessment criteria, and the last hierarchy comprising assessed plans or alternatives.

Subsequently, the assessed criteria were established. A questionnaire was created to support the decisions related to the dimensions and criteria. We reviewed literature using drug value assessment frameworks from Taiwan and other countries, Health Technology Assessment (HTA) reports, and other literature about the MCDA and hierarchical analysis applications. Finally, experts were invited to evaluate the content validity of the questionnaire. The correlation and relevance assessment of the content validity was carried out by six experts from different fields. The scoring was on a four-point system, in which one or two points were deleted, and three or four points were adopted, and the questionnaire content was revised according to the expert recommendations. [27] Once the overall content validity index (CVI) was established as higher than 0.83, the expert validity was verified. [28]

Based on the literature review, expert consultation interview, and questionnaire content validity results, nine assessment criteria were set in this study. [17, 29–31] The criteria included the comparative efficacy of treatment, the comparative safety of treatment, convenience and quality of life, cost-effectiveness, the size of the patient group affected by the disease, national expenditures, degree of drug innovation, social concerns, patient needs, and insurance coverage in other countries. Among these criteria, the first three items were of clinical value, following by three items of economic value, and the last three items, which were of social value. To facilitate descriptions in the subsequent text, the criteria are abbreviated as comparative efficacy, comparative safety, convenience and quality of life, cost-effectiveness, number of patients, expenditures, degree of innovation, social concerns and patient needs, and coverage by other countries.

### 3. Weighting criteria

One of the objectives of this study is to gain insight into the assessment criteria preferences of various stakeholders (respondents). These preferences are expressed as “weights” given to the assessment dimensions and criteria, which also affect the total score of the subsequent drug value. Considering the composition of the members of the National Health Insurance Joint Decision Meeting [32], the weighting information was collected using the interview questionnaire (S1 Text), and purposive sampling was employed to screen 30 respondents, including Taiwan FDA (TFDA) officials (2 respondents), National Health Insurance Administration (NHIA) officials (3), experts and scholars (8), patient group representatives (3), doctor representatives (3), pharmacist representatives (3), hospital representatives (5) and industrial representatives (3). Patients were not involved in this study individually. The informed consent of all subjects was given disclose the related information.

The respondents gave weights according to different attributes targeting the assessment dimensions and criteria established for the purposes of this study. For the assessment dimensions, a total score of 100 was given for a weight distribution based on three aspects: clinical, economic, and social. For the criteria weights, in the same assessment dimensions, a pairwise comparison was carried out between two criteria. [25, 29] Additionally, for the assessment scale in the hierarchical analysis, the absolute values were converted into a ratio scale. During the assessment criteria weight calculation process, the “normalization of the mean of the row vectors” served as the reference for calculating the matrix approximation value. [29] Since most matrices are not consistent, this calculation method provided better accuracy than other alternatives. Ultimately, the test respondents’ answers showed consistency, where the consistency ratio (CR) passes the standard when it is less than 0.1, meaning the results demonstrate consistency. [33]

### 4. Measurement of performance

The drug performance information for each criterion included the clinical trial literature on the five targeted therapies [34–48], the clinical treatment guide for colon and rectal cancer from the National Comprehensive Cancer Network (NCCN) [4, 5], targeted therapy instruction leaflets, 2013 and 2014 Taiwan cancer registration reports [34], the top ten drug price reference countries’ insurance drug prices for the current targeted therapies[34–40], US FDA [41] and TFDA drug permit search websites [42], the Taiwan National Health Insurance reimbursement policy [49], health technology assessment report search websites [43], etc. The assessment criteria performance matrix is shown in Table 1.

**Table 1:** Criteria, data sources, assumptions and calculations.

Dimension	Criteria	Factors	Measurement unit	Data and sources	Assumptions / Calculations
1. Clinical	1.1 Comparative efficacy	1. Overall survival period	Months	Clinical trial	“head-to-head” comparison or not for first-line clinical situation use or not hazard ratios are significant or not
		2. Progression-free survival period	Months	Clinical trial	
	1.2 Comparative safety	1. Overall incidence of adverse events	Percentage (%)	Clinical trial	-
		2. Incidence of adverse events (over Grade 3)	Percentage (%)	Clinical trial	-
		3. Dosage adjustment for special groups	Need adjustment (Yes/No)	Instruction leaflet	-
		4. Drug-drug interaction	Yes/No	Instruction leaflet	-
	1.3 Convenience and quality of life	1. Formulation	Oral / Injection	Instruction leaflet	-
		2. Frequency of use	Frequency of use	Instruction leaflet	-
		3. Combined chemotherapy prescription	With/without impacts	Clinical trial	-
		4. Treatment duration	Month	Clinical trial	-
		5. Quality of life	With/without impacts	Physician’s experience	-
2. Economic	2.1 Cost-effectiveness	ICER	No unit +/+: high cost / long progression-free survival +/-: high cost / short progression-free survival -/+: low cost / long progression-free survival -/-: low cost / short progression-free survival	Cost: International pharmaceuticals price Treatment dose: Instruction leaflet Benefit: Clinical trial	ICER = Δmonthly target therapy cost / Δtime to disease progression Compare medicines with similar status (ex. the same line of treatment) Consider the cost for side effect treatment
	2.2 Number of patients	1. Number of patients who would use this medicine	Number of patients	Taiwan Cancer registry report	Consider gene-type variance ratios and clinical treatment options
		2. Indications	With/without impacts	Instruction leaflet	-
	2.3 Expenditures	Overall target therapy expenditures	NT dollars	Fees: International pharmaceutical prices Number of people: Cancer registry reports Treatment duration: Clinical trials	Overall expenditures = number of patients * treatment cost * duration Count total costs
3. Society	3.1 Degree of innovation	1. Approval time by Taiwan FDA and US FDA	Calendar year	US FDA Website Taiwan FDA website,	-
		2. Mechanism	With/without innovation	Instruction leaflet	-
	3.2 Social concerns and patient needs	1. Irreplaceability	With/without alternative drugs	Reimbursement policy by National Health Insurance	Consider the genetic mutation situation
		2. Self-paid	Yes/No	Reimbursement policy by National Health Insurance	-
	3.3 Coverage by other countries	1. Recommendation for the coverage by Health Technology Assessment (HTA) Reports	Recommended or not	health technology assessment (HTA) reports	For first- and second-line treatments
		2. Countries offering insurance coverage for the drugs	Coverage or not	International pharmaceutical prices	-

**References**: Clinical Trials [34–48]; clinical treatment guidelines [4, 5]; International pharmaceutical prices [34–40]; Taiwan cancer registration reports [34]; US FDA website [41] and Taiwan FDA websites [42], Taiwan National Health Insurance reimbursement policy [49], health technology assessment report [43]

### 5. Scoring alternatives

An interview questionnaire (S2 Text) was adopted in this study to collect the clinical experts’ scores for the respective targeted therapies based on the respective criteria. First, the scoring scale was set to range from 1 to 5. Then, according to the score criteria definitions, the meanings of the high and low scores (See S1 Table for scoring standard detail) were determined. Purposive sampling was subsequently used to select suitable interview subjects, including colorectal cancer surgeons, hematology division doctors specialized in colorectal cancer treatment, and clinical pharmacists for cancer patients, providing 10 subjects in total. The study was conducted in accordance with the protocol approved by the Institutional Review Board (IRB) of National Cheng Kung University Hospital under IRB number B-ER-105-400.

### 6. Calculating aggregate scores

We calculated the aggregate scores for each medicine using the dimension and criteria weights, which showed the performance of each medicine as it related to each criterion. Finally, the overall score of the targeted therapies and the scores for each criterion were calculated to show the multiple values of the targeted therapies.

### 7. Dealing with uncertainty

By adjusting the assessment dimensions or criteria weights, the robustness of the research results could be confirmed. Data uncertainty affects research design and data evidence assessment results. A sensitivity analysis was conducted to evaluate the robustness of the final results. The weights of the respective assessment dimensions were adjusted as necessary, and the weights of the criteria with the top three weights were adjusted. The adjustment range of the dimensions and criteria was the upper and lower limit of the 95% confidence level.

### 8. Interpretation and reporting the results

The MCDA results present the total overall score, the scores for the dimensions, and the scores for the criteria by means of a table or a cumulative bar chart. The score information further serves as a reference for future determination of resource allocation, hospital drug entry strategies, clinical drug treatment choices, and the directions for R&D for pharmaceutical companies, thus helping decision-makers.

## Results

An expert validity assessment was conducted on the definitions and measurement methods used for the questionnaire dimensions, criteria, and weights. This assessment is conducted to determine if an assessment measures what it is intended to measure. The relevance and appropriateness of the measurement method of the dimensions and criteria had coefficients of 1; the relevance and appropriateness of the weight comparison method had reliability coefficients of 1 and 0.958, respectively, which is higher than the suggested 0.83. Hence, the expert validity assessment standard was reached.

**Fig 1:**
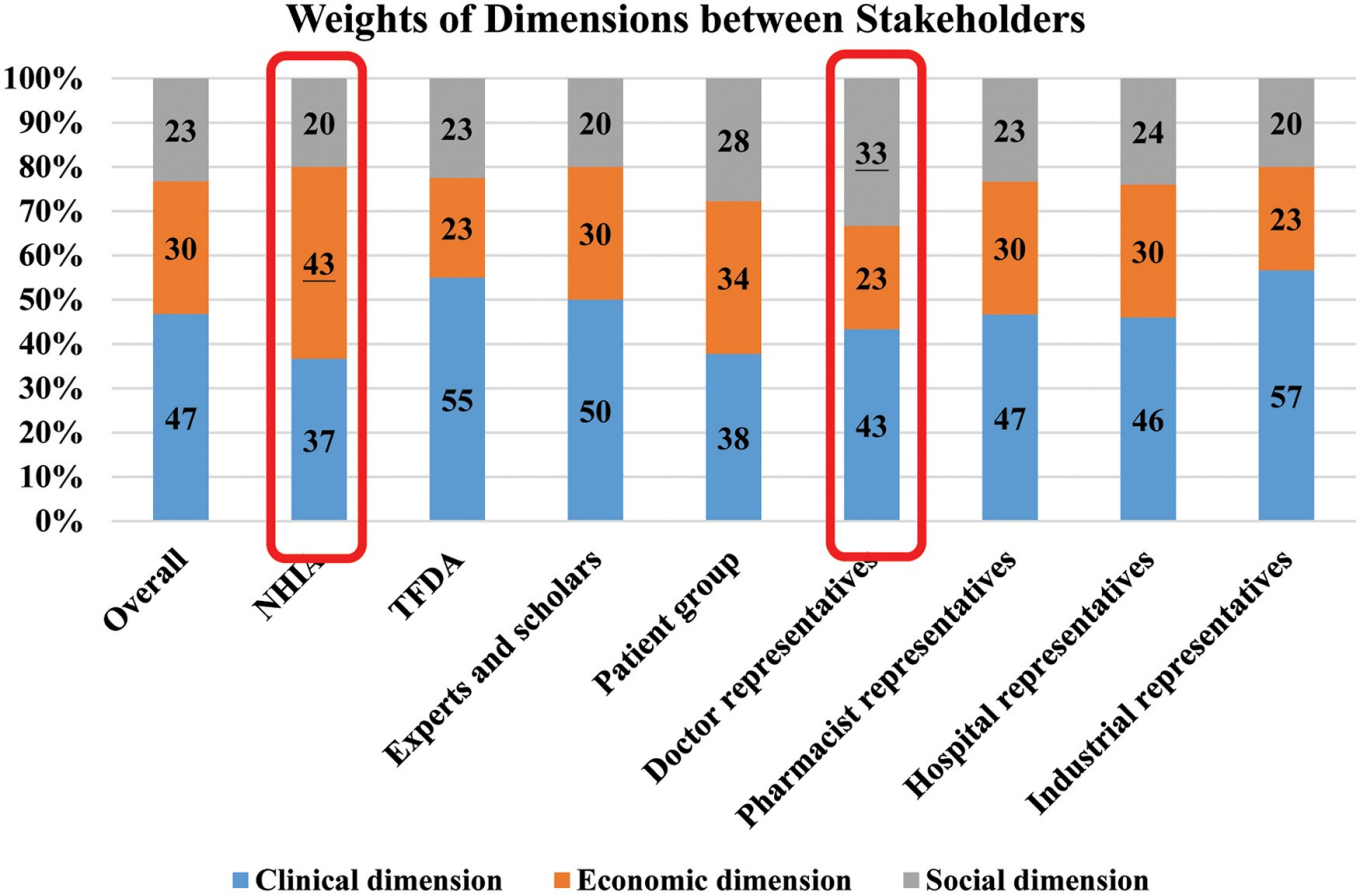
Weight of dimensions between stakeholders.

The weights of the dimensions in “dimension weights” from high to low were as follows: the clinical, economic, and social dimensions (S2 Table, Table 2 and Fig 1). After further analysis of the different respondent attributes, the dimension weight distribution could be divided into the following three types: (1) clinical dimension > economic dimension, (2) economic dimension > clinical dimension, and (3) social dimension > economic dimension. Specifically, the attributes of the respondents comprising TFDA, experts and scholars, patient groups, pharmacists, hospitals, and industrial representatives were categorized as the first type; the NHIA representatives were placed in the second type, with the economic dimension weight accounting for about 43.3%, and the physicians were categorized as the third type, with the social dimension weight consideration accounting for about 33.3%.

**Table 2:** Weights of overall and different stakeholder preferences on the criteria.

Stakeholders	Mean Weight and Ranking	Dimensions			Criteria								
		1. Clinical	2. Economic	3. Social	1. Clinical Dimension			2. Economic Dimension			3. Social Dimension		
					1.1 Efficacy	1.2 Safety	1.3 Convenience and life quality	2.1 Cost effectiveness	2.2 Number of patients	2.3 Drug costs	3.1 Degree of innovation	3.2 Patient needs	3.3 Coverage by other countries
**Overall**	**Mean Weight (95% C.I.)**	0.468	0.299	0.233	0.217	0.154	0.096	0.110	0.096	0.094	0.064	0.104	0.066
		(0.360, 0.576)	(0.200, 0.399)	(0.147, 0.319)	(0.200, 0.234)	(0.141, 0.168)	(0.082, 0.110)	(0.098, 0.122)	(0.081, 0.111)	(0.079, 0.109)	(0.055, 0.072)	(0.094, 0.113)	(0.056, 0.075)
	**Ranking**	1	2	3	1	2	5	3	5	7	9	4	8
**National Health Insurance Administration**	**Mean Weight**	0.367	0.433	0.200	0.183	0.110	0.073	0.159	0.104	0.171	0.043	0.070	0.087
	**Ranking**	2	1	3	1	4	7	3	5	2	9	8	6
**Food and Drug Administration**	**Mean Weight**	0.550	0.225	0.225	0.228	0.228	0.094	0.073	0.101	0.051	0.048	0.113	0.064
	**Ranking**	1	2	2	1	1	5	6	4	8	9	3	7
**Experts/Scholars**	**Mean Weight**	0.500	0.300	0.200	0.253	0.148	0.100	0.123	0.072	0.105	0.070	0.068	0.062
	**Ranking**	1	2	3	1	2	5	3	6	4	7	8	9
**Patients Group**	**Mean Weight**	0.378	0.344	0.278	0.157	0.107	0.113	0.107	0.054	0.184	0.074	0.130	0.074
	**Ranking**	1	2	3	2	5	4	5	9	1	7	3	7
**Physicians**	**Mean Weight**	0.433	0.233	0.333	0.213	0.137	0.083	0.092	0.079	0.062	0.086	0.194	0.054
	**Ranking**	1	3	2	1	3	6	4	7	8	5	2	9
**Pharmacists**	**Mean Weight**	0.467	0.300	0.233	0.214	0.133	0.119	0.096	0.121	0.082	0.062	0.125	0.046
	**Ranking**	1	2	3	1	2	5	6	4	7	8	3	9
**Hospitals**	**Mean Weight**	0.460	0.200	0.240	0.196	0.167	0.098	0.096	0.128	0.076	0.051	0.110	0.079
	**Ranking**	1	3	2	1	2	5	6	3	8	9	4	7
**Industrialists**	**Mean Weight**	0.567	0.233	0.200	0.283	0.170	0.113	0.083	0.075	0.075	0.057	0.100	0.043
	**Ranking**	1	2	3	1	2	3	5	6	6	8	4	9

95% C.I. = 95% confidence level

**Fig 2:**
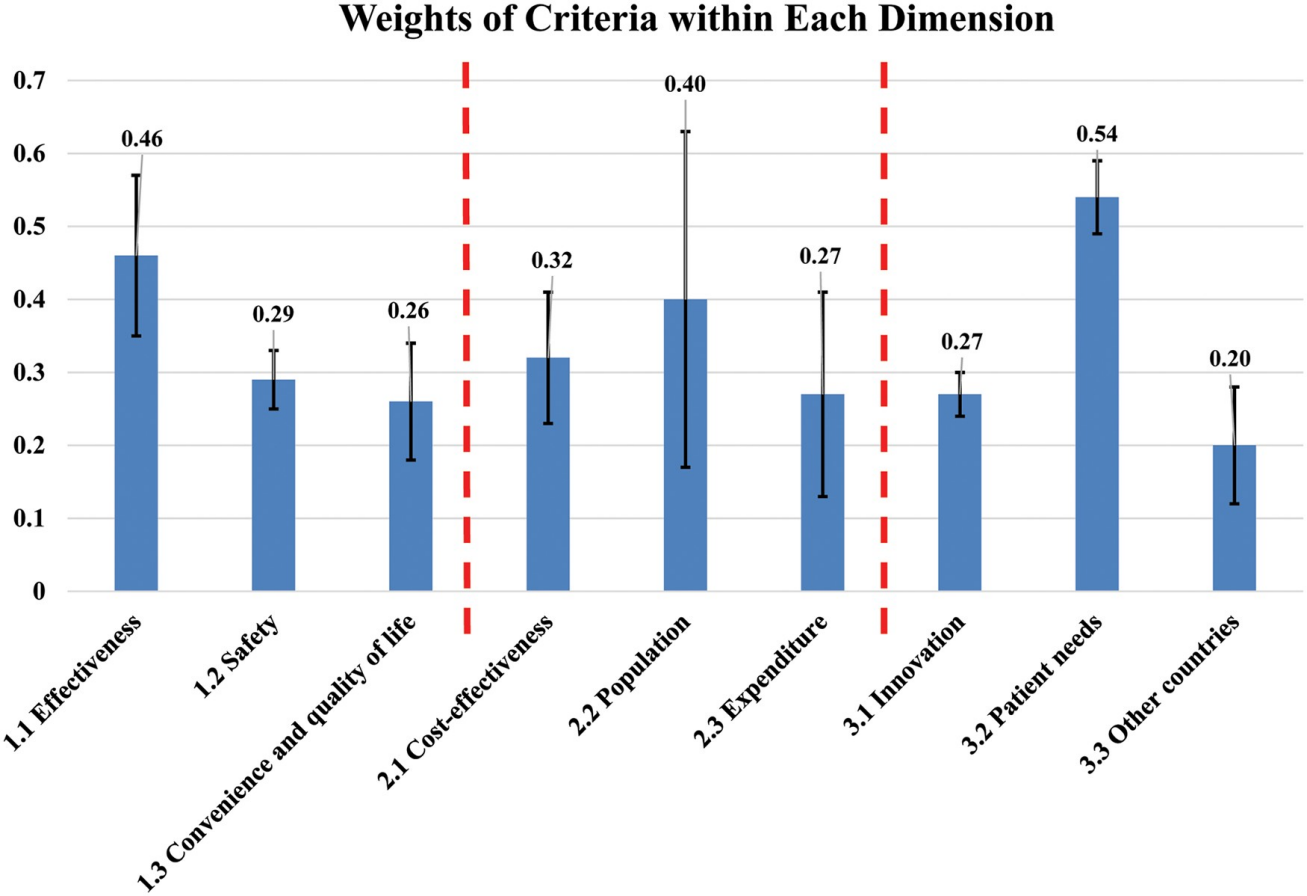
Weight of criteria within each dimension.

Overall, for the clinical dimension, the weights of criteria from high to low were: comparative efficacy, comparative safety, convenience and quality of life criteria; in the economic dimension, they were: cost-effectiveness, number of patients, and expenditures criteria weight proximity; in the social area, social concern and patient needs criteria had the highest weights, followed by degree of innovation and coverage by other countries (Table 2 and Fig 1). After excluding the answers in the clinical, economic, and social dimension that failed to pass the consistency test, the dimension and criteria weights were multiplied to obtain the cross-dimensional weight distribution of all of the assessed criteria. Therefore, the nine assessed criteria added together equaled 1. Overall, the efficiency criterion had the highest weight, followed by the safety criterion, with the cost-effectiveness criterion ranking third (Table 2 and Fig 2).

In terms of the comparative efficacy criterion, among the EGFR inhibitors, cetuximab and panitumumab had higher scores; for the comparative safety criterion, the scores of the five targeted therapies did not differ to any significant degree; for the convenience and quality of life criterion, cetuximab had the lowest score, while bevacizumab and regorafenib had the highest scores; for the cost-effectiveness criterion, cetuximab had the highest score; for the number of patients criterion, bevacizumab had the greatest number of users; for the drug expenditure criterion, bevacizumab had the highest drug expenditures, followed by cetuximab; for the degree of drug innovation criterion, regorafenib had the highest score; for the social concern and patient needs criteria, bevacizumab and regorafenib had the highest scores; finally, for the coverage by other countries, bevacizumab and cetuximab had the highest scores (S3 and S4 Tables).

The overall values of the five targeted therapies were calculated based on the weights and performance (Table 3). Overall, bevacizumab, cetuximab, and panitumumab had the highest values, while aflibercept and regorafenib had the lowest values. For the comparative efficacy criterion, cetuximab had the highest score, followed by panitumumab; for the comparative safety criterion, the scores of the five targeted therapies did not differ to any great degree, with cetuximab being the highest; for the convenience and quality of life criterion, bevacizumab and regorafenib had the highest scores; for the number of patients criterion, bevacizumab had the highest number of users; for the drug expenditure criterion, bevacizumab was found to be the most costly. The perspective of the NHIA was used to consider the resource distribution. The lower the score for expenditure criterion, a lower expenditure required by the criterion resulted in a higher value. Hence, regorafenib had the highest value in the criterion; for the degree of innovation criterion, regorafenib had the highest score; for the social concern and patient need criterion, bevacizumab had the highest score, followed by regorafenib and cetuximab; finally, for the coverage by other countries, bevacizumab and cetuximab had the highest scores.

**Table 3:** Overall scores for the targeted therapies.

Criteria	Score and Ranking	Bevacizumab	Cetuximab	Panitumumab	Aflibercept	Regorafenib
**Overall Scores**	**Score**	3.3043	3.3666	3.2030	2.8923	2.8366
	**Ranking**	2	1	3	4	5
**1. Clinical dimension**						
**1.1 Comparative efficacy**	**Score**	0.7161	0.8680	0.8246	0.6293	0.4774
	**Ranking**	3	1	2	4	5
**1.2 Comparative safety**	**Score**	0.4774	0.5082	0.5236	0.4774	0.4774
	**Ranking**	3	2	1	3	3
**1.3 Convenience and quality of life**	**Score**	0.3456	0.2592	0.3168	0.3072	0.3456
	**Ranking**	1	5	3	4	1
**2. Economic dimension**						
**2.1 Cost-effectiveness**	**Score**	0.3740	0.4400	0.3850	0.3410	0.2200
	**Ranking**	3	1	2	4	5
**2.2 Number of patients**	**Score**	0.4704	0.3648	0.2592	0.1920	0.2112
	**Ranking**	1	2	3	5	4
**2.3 Expenditures**	**Score**	0.0000	0.0940	0.2068	0.3290	0.2632
	**Ranking**	5	4	3	1	2
**3. Social dimension**						
**3.1 Degree of innovation**	**Score**	0.1984	0.1856	0.1856	0.2368	0.2496
	**Ranking**	3	4	4	2	1
**3.2 Social concerns and patient needs**	**Score**	0.4056	0.3432	0.2704	0.2080	0.3744
	**Ranking**	1	3	4	5	2
**3.3 Coverage by other countries**	**Score**	0.3168	0.3036	0.2310	0.1716	0.2178
	**Ranking**	1	2	3	5	4

After adjusting the weights of the clinical, economic, and social dimensions, the dimensions within the upper and lower boundary at a 95% confidence level underwent sensitivity testing. The results showed that there was no effect on the value ranking of the targeted therapies. However, after the social dimension was adjusted, there was a greater effect on the overall scores for bevacizumab and regorafenib. As the weight of the social dimensions increased, the value ranking of bevacizumab and regorafenib approximated that of cetuximab and aflibercept, nearly reaching consistency. After adjusting the weights of the comparative efficacy, comparative safety, and cost-effectiveness criteria, the criteria within the upper and lower boundary at a 95% confidence level underwent sensitivity testing. The results showed there to be no effect on the targeted therapy ranking, and the change caused no significant changes in the overall scores for the targeted therapies.

## Discussion

Drug value evaluations should consider multiple dimensions and criteria. [50, 51] The NHI formulary listing of drugs often takes into consideration the comparative efficacy, comparative safety, cost-effectiveness, and overall expenditures. However, the results showed that if only the four criteria above are considered, only 57.5% of the weights are covered. In other words, the remaining criteria (accounting for 42.5%) should be considered. In addition, when all the criteria in this study were evaluated, cetuximab had the highest overall value, but if only the four criteria above were evaluated, panitumumab had the highest overall value. Therefore, the completeness of assessment criteria will clearly affect the final values obtained in assessment results. Hence, multiple dimensions and multiple criteria should be considered when giving drug value assessment suggestions.

Similarly, opinions from multiple stakeholders should also be considered during drug value assessments. [52] Among these, the patients’ voice and the opinion of the patient group should be given more consideration than is typically the case. [53] Based on the research results, regardless of whether or not the opinions of the patient group or the industry on weights were considered, the final research results remained unaffected. Even so, diverse opinions should make it possible to arrive at more objective, consistent answers.

Weights were used to integrate the assessment dimensions and criteria in this study. The results show that the weights of the respective assessment criteria varied. Provided the weight differences of the criteria were not considered and that the nine criteria all had the same weight (0.111), the value assessment results ranked from high to low drug value; in other words, bevacizumab, cetuximab, panitumumab, regorafenib and aflibercept exhibited significant variations. Hence, it is suggested that during sample value assessments, impacts arising from “weights” should be considered in order to obtain information closer to the actual situation being considered.

The research findings showed that among all the dimensions, the clinical dimension had the highest weight, followed by the economic dimension and finally, the social dimension. The most direct considerations of the clinical dimension included comparative efficacy and comparative safety. Simultaneously, patient convenience and level of quality of life was considered to choose an appropriate treatment method. Therefore, as a result of clinical observations, considerations, and reviews of empirical literature, relevant specialty medical associations have mentioned some drug names for inclusion in the NHI formulary listing. Further, in view of the economic impact, the NHIA is most concerned about drug prices and the allowable budget. Patients, on the other hand, are mainly concerned about whether or not their treatment is covered by the NHI. If so, an increase in drug selection will not add a burden to patients; if drugs are self-paid items, patients will be financially burdened, which will in turn affect their willingness to choose the drugs and the duration of drug use. The social dimension had a lower weight. Some possible reasons for this include the fact that assessments rarely contain clear, quantified criteria, and traditionally, clinical and economic dimensions are prioritized. Nevertheless, the social dimension considerations include the patients’ level of demand for the drug, level of innovation of the drug, and if the drug can be easily replaced. Related issues have gradually begun to receive scholarly attention. As a result, this study also took into account the impacts of the social dimension. In addition to the clinical and economic dimensions, the social dimension was included to carry out multi-dimensional assessments.

According to the research results, cetuximab had the highest overall drug value. Current comparisons of approved drug prices mainly use cetuximab as the reference standard (referring to most clinical trial designs). In addition, cetuximab is the first drug to have been included in the NHI coverage. Therefore, cetuximab is also the most frequently used control item in the therapeutic dosage method. Bevacizumab had the second highest overall score, which was slightly lower than that of cetuximab. However, the scores for the therapeutic effectiveness of bevacizumab, number of patient users, patient demand, and other criteria all show its strengths. As for panitumumab use, its competitor is an EGFR inhibitor (cetuximab) that is similar in nature. According to the NHI provisions for drug use, panitumumab use makes it an alternative to cetuximab, which decreases the number of users of cetuximab. Aflibercept has not yet been included in Taiwan’s NHI coverage. Therefore, the financial burden on patients related to its use one of the important factors to consider. After evaluating the overall value scores, it was found that due to fewer users and the lightening of the financial burden on NHI, aflibercept may very well be an alternative therapeutic plan for patients after its inclusion in the NHI coverage. Lastly, although the overall value assessment score for oral drug regorafenib was the lowest, it was important in the individual criteria. The impact of oral drugs on convenience and quality of life was found to be negligible, especially when syringe-targeted therapies and chemotherapeutic treatment are unavailable. Thus, it can be used as an alternative treatment plan.

In recent years, MCDAs have been utilized in HTA in the fields of medicine and public health because they allow government agencies to consider the effects of effectiveness, safety, and cost of medical technology while conducting a systematic scientific empirical assessment intended to support appropriate decisions on health care payments. [54] In terms of the order of the MCDA steps, this study referred to the MCDA guidelines developed by ISPOR. [25, 29], where a value assessment model with multiple criteria for appraisal and reimbursement was established to compare the attitudes of different stakeholders toward various dimensions and criteria and to evaluate the integrated value of five targeted therapies for metastatic colorectal cancer. The steps taken in this study were similar to those set forth in the ISPOR guidelines [29] except that the order of some of the items was slightly different. Followed by structuring the criteria, the scheduling used in this study involved logical thinking. We first developed the weighting criteria, and then the measurement of performance and scoring alternatives, where the ISPOR guidelines were used first to set up the performance measurement and the scoring alternatives, followed by the weighting criteria. [29] In terms of the weighting of criteria, the “pairwise” method recommended by ISPOR guidelines was used in this study. However, in order to ensure the consistency of the respondents’ answers, a consistency ratio test [33] was also used to exclude inconsistent data to make the weighting results more accurate.

As far as the selection of criteria, three dimensions (clinical, economic and social) were used to cover nine criteria for evaluation. Angelis and Kanavos (2017) [55] proposed a framework with five dimensions (burden of disease, therapeutic, safety, innovation, and socioeconomic) to cover 14 criteria (severity, availability, prevalence, direct endpoints, surrogate endpoints, adverse events, tolerability, contraindications, clinical novelty, nature of treatment, ease of use & comfort, public health, budget impact and social productivity). The criteria for the current study covered most of the previous criteria, but because this study focused on treatments for colorectal cancer, which is a single serious disease, the burden of disease and social productivity were relatively unimportant. However, “coverage by other countries” was also considered because the decision-making context set in this study was a single country (Taiwan), where the decision-making process can assist HTAs and the determination of insurance payments.

Angelis and Montibeller (2017) used the MCDA method to evaluate the value of colorectal cancer drugs. [56] They used four dimensions (therapeutic, safety, innovation and socioeconomic) to cover 14 criteria (direct endpoints, indirect endpoints, adverse events, contraindications, mechanism of action, spill-over effect, patient convenience and direct costs). Their work specifically emphasized the innovation dimension and used a mechanism of action (the technology’s relative market entrance in regards to its ATC), spill-over effect (the number of new indications for which the technology is investigated in clinical trials), marketing authorization (the number of new indications for which the technology has gained a marketing authorization approval) and posology (the frequency of doses in a given time period in combination with the duration of the administration). In order to avoid paying too much attention to a single criteria, a relatively simple way was used to measure drug innovation: mechanism and approval time by the US FDA and the Taiwan FDA. In addition, regarding the economic dimension, only direct costs were used in Angelis and Montibeller’s work, but this study added number of patients and cost-effectiveness criteria; for the social dimension, “social concerns and patient need” and “coverage by other countries criteria were added in the present study. This arrangement allowed for more complete information related to the judgment of the economic and social dimensions. In addition, Angelis and Montibeller (2017) [56] compared three drugs, cetuximab, panitumumab and afliberce, and their results showed that the overall value of the three drugs were ranked in the following order: cetuximab, panitumumab, and then aflibercept. The results of this study are consistent with the previous results. Furthermore, Angelis and Montibeller (2017) only invited 13 decision conference participants to determine the decision model, while in the current study 30 respondents were included, and the difference in the weights of the different attributes of the stakeholder for the various dimensions (see Fig 1) were analyzed.

This study is one of the few studies in Taiwan to make use of a user MCDA to study respondents’ attribute preferences, especially using existing targeted therapies for metastatic colorectal cancer as examples to integrate the current clinical drug information and use situation. The study further quantitatively presented the drug values using academic methods, which enables insurance authorities and communities to determine both the overall and classified values of drugs. Therefore, this study is a contribution to both the academic community and to practice.

This study has a number of limitations. First, the related referenced information obtained changed over time. For example, the reference information in this study was obtained by compiling actual attainable information, which was provided as a reference for the experts and respondents in the study. However, as medical technology follows its current trend toward rapid progress, there will be new drugs developed and launched in the future that will be covered by NHI, and newer drug-related information will be available. Therefore, the drug value assessment model should be adjusted dynamically on a timely basis. Secondly, due to the limited available literature on this topic, some of the assessment criteria reference information may not be entirely accurate or complete. For example, it was impossible to provide measurements of the quality of life related to the various types of drugs under consideration in this study. In this regard, it is suggested that respondents (medical experts) make overall judgments based on general quality of life-related indicators based on their own clinical drug use experience. Furthermore, this study evaluated the drug values from the perspective of global public sector national health insurance institutions, so the drug-related information cited is based on international clinical trial results, clinical trial guides, drug instruction leaflet information, international median drug prices, and so on. In the future, local efficacy, safety, and cost-effectiveness analyses may be carried out in advance before using this research framework to carry out local drug price assessment studies.

## Conclusion

In this study, a set of multi-dimensional assessment decision models was established through a compilation of empirical data literature. Additionally, an interview questionnaire method was employed to gain insight into the respondents’ attribute preferences and to further quantitatively the presented values. It was found in the study that among the many multiple assessment dimensions and criteria, regardless of the respondents’ attributes, the comparative efficacy criterion for the clinical dimension was the primary consideration factor. This finding suggests that after taking into account the importance of the decision group related to the assessment criteria, in terms of the analytical results for the targeted therapies for metastatic colorectal cancer, cetuximab was shown to have the highest combined value. The rest of the drugs as ranked by value in the following order: bevacizumab, panitumumab, aflibercept, and regorafenib. Overall, this study used “multiple dimensions,” “multi-perspectives,” and “integration” as starting points. The MCDA method was used to comprehensively evaluate the value of the drugs under consideration. In addition, five existing targeted therapies for metastatic colorectal cancer were adopted to carry out the pilot study. The research results should serve as a basis for relevant follow-up studies or as a reference for future drug R&D management, drug launch management, NHI policy decision-making, hospital drug introduction and procurement, clinical drug use, and other practices in respective fields, thereby further achieving effective resource distribution and use.

## Supporting information

S1 Text Questionnaire: Weighting of dimensions and criteria. (DOCX)

S2 Text Questionnaire: Scoring of treatments. (DOCX)

S1 Table Scoring standards for each criteria. (DOCX)

S2 Table Data for weights of overall and different stakeholder preferences on the criteria. (DOCX)

S3 Table Assessment scores of targeted therapies by criteria. (DOCX)

S4 Table Data for scoring of treatments. (DOCX)
