# Supplementary material for: Comprehensive value assessment of drugs using a multi-criteria decision analysis: An example of targeted therapies for metastatic colorectal cancer treatment
Source: PLoS One. 2019 Dec 12;14(12):e0225938. doi: 10.1371/journal.pone.0225938 (PMC6907782; doi:10.1371/journal.pone.0225938)
Supplement: S1 Text — (DOCX) [file pone.0225938.s001.docx]

**S1 Text. Questionnaire: Weighting of Dimensions and Criteria**

Dear Expert:

This questionnaire is constructed based on the value evaluation model of the target therapies for colorectal cancer treatment. Its purpose is to lead to an understanding of the relative importance of each criterion when measuring the value of the given medicine. The criteria listed in the questionnaire are shown in the table below. We invite you to evaluate the relative importance of the criteria based those listed in the questionnaire and to provide suggestions related to the content.

- **Criteria and measurements**

| **Dimensions and Criteria** | **Definition / Measurement** |
| --- | --- |
| **1. Clinical dimension** | |
| 1.1 Comparative efficacy | This refers to the treatment medians for overall survival and progression free survival compared with the control group, which were used to represent the effect of the target therapies. |
| 1.2 Comparative safety | This refers to adverse drug reactions and their incidences in the target therapies, special ethnic groups (elderly, liver/kidney insufficiency, and pregnancy and lactation), as well as to drug interactions (reference drug or clinical trial literature). |
| 1.3 Convenience and quality of life | This refers to the dosage form of the drug, the frequency of use, whether it is combined with chemotherapy, the estimated course of treatment, and the EQ-5D and EORTC QLQ-C30, which measure the quality of life of patients. |
| **2. Economic dimension** | |
| 2.1 Cost-effectiveness | Incremental cost-effectiveness ratio (ICER) |
| 2.2 Number of patients | This refers to the number of patients who are estimated to be using the target drugs, taking into consideration the contraindications of the drug and the number of patients who may be using the drug. |
| 2.3 Expenditures | National targeted drug expenditure = “the estimated course cost for each patient using the target therapies” * “the total number of people using the target therapies” |
| **3. Social dimension** | |
| 3.1 Degree of innovation | This criterion considers the time at which the drug was approved in Taiwan, as well as the mechanism and breakthroughs in the efficacy of the drugs (based on the clinical evidence). |
| 3.2 Social concerns and patient needs | This refers to the choices of other clinically available drugs (representing the irreplaceability of drugs) and other special circumstances. |
| 3.3 Coverage by other countries | This refers to the insurance coverage of the medicine in other advanced countries such as the UK, Australia, Canada, and Scotland. |

- **Scoring criteria for the relative importance of criteria:**

1: equally important

2: somewhat more important

3: much more important

| Left Criterion is more important |  | | | | | Right Criterion is more important |
| --- | --- | --- | --- | --- | --- | --- |
|  | 3 | 2 | 1 | 2 | 3 |  |
| 1.1 Comparative efficacy | ○ | ○ | ● | ○ | ○ | 1.2 Comparative safety |

**Part 1. Dimensions**

Please give scores with the following three dimensions: clinical, economic, and social. The total possible score is 100.

**Clinical dimension: Economic dimension: Social dimension = _____: _____: _____**

**Part 2. Clinical dimension**

| Left Criterion is more important |  | | | | | Right Criterion is more important |
| --- | --- | --- | --- | --- | --- | --- |
|  | 3 | 2 | 1 | 2 | 3 |  |
| 1.1 Comparative efficacy | ○ | ○ | ○ | ○ | ○ | 1.2 Comparative safety |
| 1.1 Comparative efficacy | ○ | ○ | ○ | ○ | ○ | 1.3 Convenience and quality of life |
| 1.2 Comparative safety | ○ | ○ | ○ | ○ | ○ | 1.3 Convenience and quality of life |

**Part 3. Economic dimension**

| Left Criterion is more important |  | | | | | Right Criterion is more important |
| --- | --- | --- | --- | --- | --- | --- |
|  | 3 | 2 | 1 | 2 | 3 |  |
| 2.1 Cost-effectiveness | ○ | ○ | ○ | ○ | ○ | 2.2 Number of patients |
| 2.1 Cost-effectiveness | ○ | ○ | ○ | ○ | ○ | 2.3 Expenditures |
| 2.2 Number of patients | ○ | ○ | ○ | ○ | ○ | 2.3 Expenditures |

**Part 4. Social dimension**

| Left Criterion is more important |  | | | | | Right Criterion is more important |
| --- | --- | --- | --- | --- | --- | --- |
|  | 3 | 2 | 1 | 2 | 3 |  |
| 3.1 Degree of innovation | ○ | ○ | ○ | ○ | ○ | 3.2 Social concerns and patient needs |
| 3.1 Degree of innovation | ○ | ○ | ○ | ○ | ○ | 3.3 Coverage by other countries |
| 3.2 Social concerns and patient needs | ○ | ○ | ○ | ○ | ○ | 3.3 Coverage by other countries |

**Part 5. Other Suggestions for the questionnaire design**

|  |
| --- |

**◎ Basic Personal Information:**

**1. Category**

□ Government authority

□ National Health Insurance representative

□ Representative of experts and scholars

□ Clinical Medicine

□ Clinical Pharmacy

□ Pharmacoeconomics

□ Patient group representative

□ Industry representative

**2. Institute**

□ National Health Insurance Administration

□ Taiwan Food and Drug Administration

□ Center for Drug Evaluation, Taiwan

□ Hospital

□ University

□ Pharmaceutical company

□ Cancer Foundation

□ Others:_______________

**3. Years of Service**

□<1 year □1-5 years □6-10 years □11-15 years □16-20 years □> 21 years

**4.** Age

□21-30 □31-40 □41-50 □>51

**5. Gender**

□ Male □ Female

**6. Highest Level of Education**

□ Undergraduate □ Master □ Ph.D.
