# Supplementary material for: Comprehensive value assessment of drugs using a multi-criteria decision analysis: An example of targeted therapies for metastatic colorectal cancer treatment
Source: PLoS One. 2019 Dec 12;14(12):e0225938. doi: 10.1371/journal.pone.0225938 (PMC6907782; doi:10.1371/journal.pone.0225938)
Supplement: S2 Text — (DOCX) [file pone.0225938.s002.docx]

**S2 Text. Questionnaire: Scoring of Treatments**

Dear Expert:

This questionnaire was constructed based on the value evaluation model of the target therapies for colorectal cancer treatment. Its purpose is to determine the relationship between the relative scores for each treatment and each criterion, for which the relevant criteria are in the table below. We invite you to evaluate the relative scores for the treatments. If you have any suggestions related to the questionnaire content, please let us know. Thank you very much.

PS. Score 1: the worst; score 5: the best.

1. **Clinical Dimension**

| **Criterion 1.1 Comparative efficacy** | | | | | | | | | | | | | | | | | | | | | | | | |
| --- | --- | --- | --- | --- | --- | --- | --- | --- | --- | --- | --- | --- | --- | --- | --- | --- | --- | --- | --- | --- | --- | --- | --- | --- |
| Based on the drugs’ overall survival and progression free survival, the better the therapeutic effect, the higher the score. | | | | | | | | | | | | | | | | | | | | | | | | |
| **Bevacizumab** | | | | | **Cetuximab** | | | | | **Panitumumab** | | | | | **Aflibercept** | | | | | **Regorafenib** | | | | |
| ○ | ○ | ○ | ○ | ○ | ○ | ○ | ○ | ○ | ○ | ○ | ○ | ○ | ○ | ○ | ○ | ○ | ○ | ○ | ○ | ○ | ○ | ○ | ○ | ○ |
| **1** | **2** | **3** | **4** | **5** | **1** | **2** | **3** | **4** | **5** | **1** | **2** | **3** | **4** | **5** | **1** | **2** | **3** | **4** | **5** | **1** | **2** | **3** | **4** | **5** |

| **Criterion 1.2 Comparative safety** | | | | | | | | | | | | | | | | | | | | | | | | |
| --- | --- | --- | --- | --- | --- | --- | --- | --- | --- | --- | --- | --- | --- | --- | --- | --- | --- | --- | --- | --- | --- | --- | --- | --- |
| Based on the adverse drug reactions and their incidences of target therapies, special ethnic groups (elderly, liver/kidney insufficiency, and pregnancy and lactation), as well as drug interactions (reference drug or clinical trial literature), the better the degree of safety, the higher the score. | | | | | | | | | | | | | | | | | | | | | | | | |
| **Bevacizumab** | | | | | **Cetuximab** | | | | | **Panitumumab** | | | | | **Aflibercept** | | | | | **Regorafenib** | | | | |
| ○ | ○ | ○ | ○ | ○ | ○ | ○ | ○ | ○ | ○ | ○ | ○ | ○ | ○ | ○ | ○ | ○ | ○ | ○ | ○ | ○ | ○ | ○ | ○ | ○ |
| **1** | **2** | **3** | **4** | **5** | **1** | **2** | **3** | **4** | **5** | **1** | **2** | **3** | **4** | **5** | **1** | **2** | **3** | **4** | **5** | **1** | **2** | **3** | **4** | **5** |

| **Criterion 1.3 Convenience and quality of life** | | | | | | | | | | | | | | | | | | | | | | | | |
| --- | --- | --- | --- | --- | --- | --- | --- | --- | --- | --- | --- | --- | --- | --- | --- | --- | --- | --- | --- | --- | --- | --- | --- | --- |
| Based on the dosage form of the drug, the frequency of use, whether its use is combined with chemotherapy, the estimated course of treatment, and the EQ-5D and EORTC QLQ-C30, the more convenient and the better the quality of life, the higher the score. | | | | | | | | | | | | | | | | | | | | | | | | |
| **Bevacizumab** | | | | | **Cetuximab** | | | | | **Panitumumab** | | | | | **Aflibercept** | | | | | **Regorafenib** | | | | |
| ○ | ○ | ○ | ○ | ○ | ○ | ○ | ○ | ○ | ○ | ○ | ○ | ○ | ○ | ○ | ○ | ○ | ○ | ○ | ○ | ○ | ○ | ○ | ○ | ○ |
| **1** | **2** | **3** | **4** | **5** | **1** | **2** | **3** | **4** | **5** | **1** | **2** | **3** | **4** | **5** | **1** | **2** | **3** | **4** | **5** | **1** | **2** | **3** | **4** | **5** |

**2. Economic Dimension**

| **Criterion 2.1 Cost-effectiveness** | | | | | | | | | | | | | | | | | | | | | | | | |
| --- | --- | --- | --- | --- | --- | --- | --- | --- | --- | --- | --- | --- | --- | --- | --- | --- | --- | --- | --- | --- | --- | --- | --- | --- |
| Based on the incremental cost-effectiveness ratio (ICER), the lower the ratio (or more cost-effectiveness), the higher the score. | | | | | | | | | | | | | | | | | | | | | | | | |
| **Bevacizumab** | | | | | **Cetuximab** | | | | | **Panitumumab** | | | | | **Aflibercept** | | | | | **Regorafenib** | | | | |
| ○ | ○ | ○ | ○ | ○ | ○ | ○ | ○ | ○ | ○ | ○ | ○ | ○ | ○ | ○ | ○ | ○ | ○ | ○ | ○ | ○ | ○ | ○ | ○ | ○ |
| **1** | **2** | **3** | **4** | **5** | **1** | **2** | **3** | **4** | **5** | **1** | **2** | **3** | **4** | **5** | **1** | **2** | **3** | **4** | **5** | **1** | **2** | **3** | **4** | **5** |

| **Criterion 2.2 Number of patients** | | | | | | | | | | | | | | | | | | | | | | | | |
| --- | --- | --- | --- | --- | --- | --- | --- | --- | --- | --- | --- | --- | --- | --- | --- | --- | --- | --- | --- | --- | --- | --- | --- | --- |
| Based on the number of patients who are estimated to be using target drugs when considering the contraindications of the drug and the number of patients who may be using the drug, the greater the population, the higher the score. | | | | | | | | | | | | | | | | | | | | | | | | |
| **Bevacizumab** | | | | | **Cetuximab** | | | | | **Panitumumab** | | | | | **Aflibercept** | | | | | **Regorafenib** | | | | |
| ○ | ○ | ○ | ○ | ○ | ○ | ○ | ○ | ○ | ○ | ○ | ○ | ○ | ○ | ○ | ○ | ○ | ○ | ○ | ○ | ○ | ○ | ○ | ○ | ○ |
| **1** | **2** | **3** | **4** | **5** | **1** | **2** | **3** | **4** | **5** | **1** | **2** | **3** | **4** | **5** | **1** | **2** | **3** | **4** | **5** | **1** | **2** | **3** | **4** | **5** |

| **Criterion 2.3 Expenditures** | | | | | | | | | | | | | | | | | | | | | | | | |
| --- | --- | --- | --- | --- | --- | --- | --- | --- | --- | --- | --- | --- | --- | --- | --- | --- | --- | --- | --- | --- | --- | --- | --- | --- |
| Based on the national targeted drug expenditures, the lower the expenditures, the higher the score. | | | | | | | | | | | | | | | | | | | | | | | | |
| **Bevacizumab** | | | | | **Cetuximab** | | | | | **Panitumumab** | | | | | **Aflibercept** | | | | | **Regorafenib** | | | | |
| ○ | ○ | ○ | ○ | ○ | ○ | ○ | ○ | ○ | ○ | ○ | ○ | ○ | ○ | ○ | ○ | ○ | ○ | ○ | ○ | ○ | ○ | ○ | ○ | ○ |
| **1** | **2** | **3** | **4** | **5** | **1** | **2** | **3** | **4** | **5** | **1** | **2** | **3** | **4** | **5** | **1** | **2** | **3** | **4** | **5** | **1** | **2** | **3** | **4** | **5** |

1. **Social Dimension**

| **Criterion 3.1 Degree of innovation** | | | | | | | | | | | | | | | | | | | | | | | | |
| --- | --- | --- | --- | --- | --- | --- | --- | --- | --- | --- | --- | --- | --- | --- | --- | --- | --- | --- | --- | --- | --- | --- | --- | --- |
| Based on when the drug was approved in Taiwan, the therapeutic mechanisms, and breakthroughs in the efficacy of drugs (based on the clinical evidence), the higher the level of drug innovation, the higher the score. | | | | | | | | | | | | | | | | | | | | | | | | |
| **Bevacizumab** | | | | | **Cetuximab** | | | | | **Panitumumab** | | | | | **Aflibercept** | | | | | **Regorafenib** | | | | |
| ○ | ○ | ○ | ○ | ○ | ○ | ○ | ○ | ○ | ○ | ○ | ○ | ○ | ○ | ○ | ○ | ○ | ○ | ○ | ○ | ○ | ○ | ○ | ○ | ○ |
| **1** | **2** | **3** | **4** | **5** | **1** | **2** | **3** | **4** | **5** | **1** | **2** | **3** | **4** | **5** | **1** | **2** | **3** | **4** | **5** | **1** | **2** | **3** | **4** | **5** |

| **Criterion 3.2 Social concerns and patient needs** | | | | | | | | | | | | | | | | | | | | | | | | |
| --- | --- | --- | --- | --- | --- | --- | --- | --- | --- | --- | --- | --- | --- | --- | --- | --- | --- | --- | --- | --- | --- | --- | --- | --- |
| Based on the choices for other clinically available drugs (representing the irreplaceability of drugs) and other special circumstances, the higher the irreplaceability, the higher the score. | | | | | | | | | | | | | | | | | | | | | | | | |
| **Bevacizumab** | | | | | **Cetuximab** | | | | | **Panitumumab** | | | | | **Aflibercept** | | | | | **Regorafenib** | | | | |
| ○ | ○ | ○ | ○ | ○ | ○ | ○ | ○ | ○ | ○ | ○ | ○ | ○ | ○ | ○ | ○ | ○ | ○ | ○ | ○ | ○ | ○ | ○ | ○ | ○ |
| **1** | **2** | **3** | **4** | **5** | **1** | **2** | **3** | **4** | **5** | **1** | **2** | **3** | **4** | **5** | **1** | **2** | **3** | **4** | **5** | **1** | **2** | **3** | **4** | **5** |

| **Criterion 3.3 Coverage by other countries** | | | | | | | | | | | | | | | | | | | | | | | | |
| --- | --- | --- | --- | --- | --- | --- | --- | --- | --- | --- | --- | --- | --- | --- | --- | --- | --- | --- | --- | --- | --- | --- | --- | --- |
| Based on the insurance coverage of the medicine in other advanced countries, the greater the number of countries covering the payment for the drug, the higher the score. | | | | | | | | | | | | | | | | | | | | | | | | |
| **Bevacizumab** | | | | | **Cetuximab** | | | | | **Panitumumab** | | | | | **Aflibercept** | | | | | **Regorafenib** | | | | |
| ○ | ○ | ○ | ○ | ○ | ○ | ○ | ○ | ○ | ○ | ○ | ○ | ○ | ○ | ○ | ○ | ○ | ○ | ○ | ○ | ○ | ○ | ○ | ○ | ○ |
| **1** | **2** | **3** | **4** | **5** | **1** | **2** | **3** | **4** | **5** | **1** | **2** | **3** | **4** | **5** | **1** | **2** | **3** | **4** | **5** | **1** | **2** | **3** | **4** | **5** |

**◎ Basic Personal Information:**

**1. Category**

□ Government authority

□ National Health Insurance representative

□ Representative of experts and scholars

□ Clinical Medicine

□ Clinical Pharmacy

□ Pharmacoeconomics

□ Patient group representative

□ Industry representative

**2. Institute**

□ National Health Insurance Administration

□ Taiwan Food and Drug Administration

□ Center for Drug Evaluation, Taiwan

□ Hospital

□ University

□ Pharmaceutical company

□ Cancer Foundation

□ Others:_______________

**3. Years of Service**

□<1 year □1-5 years □6-10 years □11-15 years □16-20 years □> 21 years

**4.** Age

□21-30 □31-40 □41-50 □>51

**5. Gender**

□ Male □ Female

**6. The Highest Level of Education**

□ Undergraduate □ Master □ Ph.D.
