# Supplementary material for: Comprehensive value assessment of drugs using a multi-criteria decision analysis: An example of targeted therapies for metastatic colorectal cancer treatment
Source: PLoS One. 2019 Dec 12;14(12):e0225938. doi: 10.1371/journal.pone.0225938 (PMC6907782; doi:10.1371/journal.pone.0225938)
Supplement: S1 Table — (DOCX) [file pone.0225938.s003.docx]

**S1 Table. Scoring Standards for each Criteria**

| **Criteria** | **Positive/Negative** | **Description of Scoring** | | |
| --- | --- | --- | --- | --- |
|  |  | **1 point** | **5 point** | |
| 1.1 Comparative efficacy | Positive | The lowest degree of improvement | | The highest degree of improvement |
| 1.2 Comparative safety | Positive | The lowest safety | | The highest safety |
| 1.3 Convenience and quality of life | Positive | The least convenient and poorest quality of life | | The most convenient and best life quality |
| 2.1 Cost-effectiveness | Positive | The lowest net benefit | | The highest net benefit |
| 2.2 Number of patients | Positive | The lowest number of patients | | The highest number of patients |
| 2.3 Expenditures | Negative | The lowest drug expenditure | | The highest expenditure |
| 3.1 Degree of innovation | Positive | The least innovative | | The most innovative |
| 3.2 Social concerns and patient needs | Positive | The lowest irreplaceability | | The highest irreplaceability |
| 3.3 Coverage by other countries | Positive | Covered by the least number of countries | | Covered by the highest number of countries |
