# Supplementary material for: Comprehensive value assessment of drugs using a multi-criteria decision analysis: An example of targeted therapies for metastatic colorectal cancer treatment
Source: PLoS One. 2019 Dec 12;14(12):e0225938. doi: 10.1371/journal.pone.0225938 (PMC6907782; doi:10.1371/journal.pone.0225938)
Supplement: S2 Table — (DOCX) [file pone.0225938.s004.docx]

**S2 Table. Data for weights of overall and different stakeholder preferences on the criteria**

| **Weights** | | **Dimension** | | | **Criteria** | | | | | | | | |
| --- | --- | --- | --- | --- | --- | --- | --- | --- | --- | --- | --- | --- | --- |
| **No** | **Type of Stakeholder** | **1. Clinical** | **2. Economic** | **3.**  **Social** | **1.1 / 1.2** | **1.1 / 1.3** | **1.2 / 1.3** | **2.1 / 2.2** | **2.1 / 2.3** | **2.2 / 2.3** | **3.1 / 3.2** | **3.1 / 3.3** | **3.2 / 3.3** |
| **1** | **National Health Insurance Administration** | 30% | 60% | 10% | 3 | 3 | 2 | 2 | 3 | 2 | 1/2 | 1/2 | 2 |
| **2** | **National Health Insurance Administration** | 30% | 50% | 20% | 2 | 3 | 1/2 | 2 | 1/3 | 1/3 | 1 | 1/2 | 1 |
| **3** | **National Health Insurance Administration** | 50% | 20% | 30% | 1 | 2 | 2 | 1 | 1 | 1 | 1/2 | 1/2 | 2 |
| **4** | **Experts/Scholars** | 50% | 30% | 20% | 1 | 2 | 2 | 2 | 2 | 1 | 1 | 1 | 2 |
| **5** | **Experts/Scholars** | 50% | 30% | 20% | 2 | 3 | 3 | 2 | 1/3 | 1/3 | 1/2 | 1/2 | 1/2 |
| **6** | **Experts/Scholars** | 70% | 20% | 10% | 2 | 3 | 2 | 1/2 | 2 | 1 | 1 | 2 | 1 |
| **7** | **Experts/Scholars** | 50% | 30% | 20% | 3 | 3 | 2 | 2 | 1/2 | 2 | 2 | 1 | 2 |
| **8** | **Experts/Scholars** | 40% | 30% | 30% | 1 | 2 | 2 | 2 | 2 | 1/2 | 3 | 2 | 2 |
| **9** | **Experts/Scholars** | 40% | 30% | 30% | 2 | 2 | 1 | 2 | 1 | 1 | 2 | 2 | 2 |
| **10** | **Experts/Scholars** | 40% | 40% | 20% | 2 | 3 | 2 | 2 | 1 | 1/2 | 1 | 1 | 1 |
| **11** | **Experts/Scholars** | 60% | 30% | 10% | 2 | 2 | 3 | 1 | 3 | 3 | 1 | 1 | 1 |
| **12** | **Food and Drug Administration** | 60% | 20% | 20% | 1 | 2 | 2 | 1/2 | 1 | 2 | 1/2 | 2 | 2 |
| **13** | **Food and Drug Administration** | 50% | 25% | 25% | 1 | 3 | 3 | 1 | 2 | 2 | 1/3 | 1/3 | 2 |
| **14** | **Hospitals** | 50% | 20% | 30% | 1 | 2 | 2 | 1 | 1 | 1 | 1/2 | 2 | 2 |
| **15** | **Hospitals** | 60% | 25% | 15% | 2 | 2 | 2 | 1/2 | 1/2 | 1 | 1/3 | 1/3 | 2 |
| **16** | **Hospitals** | 50% | 30% | 20% | 1 | 2 | 2 | 1/2 | 2 | 2 | 1/2 | 1 | 2 |
| **17** | **Hospitals** | 40% | 35% | 25% | 1 | 2 | 1/2 | 2 | 2 | 1 | 1/2 | 1/3 | 1/3 |
| **18** | **Hospitals** | 30% | 40% | 30% | 1 | 2 | 2 | 1/2 | 2 | 2 | 2 | 1/2 | 2 |
| **19** | **Industrialists** | 70% | 20% | 10% | 3 | 3 | 1 | 2 | 2 | 1/2 | 1/3 | 1 | 3 |
| **20** | **Industrialists** | 50% | 30% | 20% | 2 | 3 | 1/2 | 2 | 1/2 | 1/2 | 1 | 2 | 2 |
| **21** | **Industrialists** | 50% | 20% | 30% | 1 | 2 | 2 | 1/2 | 2 | 2 | 1/2 | 1 | 2 |
| **22** | **Patient Group** | 40% | 30% | 30% | 1 | 1/2 | 1/2 | 2 | 1 | 1 | 1/3 | 1 | 3 |
| **23** | **Patient Group** | 33% | 33% | 33% | 3 | 3 | 2 | 3 | 3 | 1/3 | 1 | 1 | 1 |
| **24** | **Patient Group** | 40% | 40% | 20% | 1 | 2 | 2 | 2 | 1/3 | 1/3 | 1/2 | 3 | 2 |
| **25** | **Pharmacists** | 40% | 40% | 20% | 2 | 2 | 1 | 3 | 1 | 1/2 | 1/2 | 2 | 2 |
| **26** | **Pharmacists** | 60% | 20% | 20% | 2 | 3 | 2 | 1/2 | 1 | 2 | 1/3 | 1 | 1 |
| **27** | **Pharmacists** | 40% | 30% | 30% | 1 | 1 | 1 | 1/2 | 2 | 2 | 1/2 | 1 | 2 |
| **28** | **Physicians** | 40% | 10% | 50% | 1 | 3 | 3 | 1/2 | 2 | 2 | 1/3 | 1 | 2 |
| **29** | **Physicians** | 40% | 30% | 30% | 2 | 2 | 1 | 2 | 1 | 1 | 1/2 | 2 | 2 |
| **30** | **Physicians** | 50% | 30% | 20% | 2 | 3 | 2 | 2 | 2 | 1 | 1/2 | 2 | 3 |
