# Supplementary material for: Comprehensive value assessment of drugs using a multi-criteria decision analysis: An example of targeted therapies for metastatic colorectal cancer treatment
Source: PLoS One. 2019 Dec 12;14(12):e0225938. doi: 10.1371/journal.pone.0225938 (PMC6907782; doi:10.1371/journal.pone.0225938)
Supplement: S3 Table — (DOCX) [file pone.0225938.s005.docx]

**S3 Table. Assessment scores of targeted therapies by criteria**

| **Dimensions** | **Criteria** | **Scores (Mean ± Standard Deviation)** | | | | | | | | | | | | | | |
| --- | --- | --- | --- | --- | --- | --- | --- | --- | --- | --- | --- | --- | --- | --- | --- | --- |
|  |  | **Bevacizumab** | | | **Cetuximab** | | | **Panitumumab** | | | **Aflibercept** | | | **Regorafenib** | | |
| **1. Clinical** | **1.1 Efficacy** | 3.3 | ± | 0.67 | 4 | ± | 0.82 | 3.8 | ± | 0.63 | 2.9 | ± | 0.74 | 2.2 | ± | 1.03 |
|  | **1.2 Safety** | 3.1 | ± | 1.45 | 3.3 | ± | 0.95 | 3.4 | ± | 0.97 | 3.1 | ± | 0.88 | 3.1 | ± | 1.2 |
|  | **1.3 Convenience and life quality** | 3.6 | ± | 0.97 | 2.7 | ± | 0.95 | 3.3 | ± | 0.67 | 3.2 | ± | 0.79 | 3.6 | ± | 1.07 |
| **2. Economic** | **2.1 Cost effectiveness** | 3.4 | ± | 0.52 | 4 | ± | 0.82 | 3.5 | ± | 0.71 | 3.1 | ± | 0.99 | 2 | ± | 1.05 |
|  | **2.2 Number of patients** | 4.9 | ± | 0.32 | 3.8 | ± | 0.63 | 2.7 | ± | 1.25 | 2 | ± | 0.82 | 2.2 | ± | 0.79 |
|  | **2.3 Expenditure** | 5 | ± | 0 | 4 | ± | 0 | 2.8 | ± | 1.23 | 1.5 | ± | 0.71 | 2.2 | ± | 0.79 |
| **3. Social** | **3.1 Degree of innovation** | 3.1 | ± | 1.37 | 2.9 | ± | 1.2 | 2.9 | ± | 1.2 | 3.7 | ± | 0.82 | 3.9 | ± | 1.2 |
|  | **3.2 Patient needs** | 3.9 | ± | 1.37 | 3.3 | ± | 1.06 | 2.6 | ± | 0.52 | 2 | ± | 1.05 | 3.6 | ± | 1.26 |
|  | **3.3 Coverage by other countries** | 4.8 | ± | 0.42 | 4.6 | ± | 0.52 | 3.5 | ± | 0.53 | 2.6 | ± | 0.52 | 3.3 | ± | 1.16 |
