# Supplementary material for: Comprehensive value assessment of drugs using a multi-criteria decision analysis: An example of targeted therapies for metastatic colorectal cancer treatment
Source: PLoS One. 2019 Dec 12;14(12):e0225938. doi: 10.1371/journal.pone.0225938 (PMC6907782; doi:10.1371/journal.pone.0225938)
Supplement: S4 Table — (DOCX) [file pone.0225938.s006.docx]

**S4 Table. Data for Scoring of Treatments**

| **Score** | | **No. of subjects** | | | | | | | | | |
| --- | --- | --- | --- | --- | --- | --- | --- | --- | --- | --- | --- |
| **Criteria** | **Alternatives** | **1** | **2** | **3** | **4** | **5** | **6** | **7** | **8** | **9** | **10** |
| **1.1** | **Bevacizumab** | 2 | 3 | 4 | 4 | 3 | 3 | 4 | 4 | 3 | 3 |
| **1.1** | **Cetuximab** | 3 | 4 | 4 | 5 | 5 | 5 | 4 | 4 | 3 | 3 |
| **1.1** | **Panitumumab** | 3 | 4 | 4 | 5 | 4 | 4 | 3 | 4 | 3 | 4 |
| **1.1** | **Aflibercept** | 2 | 3 | 3 | 4 | 2 | 3 | 4 | 3 | 2 | 3 |
| **1.1** | **Regorafenib** | 1 | 3 | 2 | 2 | 1 | 3 | 2 | 3 | 1 | 4 |
| **1.2** | **Bevacizumab** | 3 | 2 | 1 | 3 | 1 | 4 | 5 | 5 | 4 | 3 |
| **1.2** | **Cetuximab** | 2 | 3 | 2 | 3 | 3 | 3 | 4 | 5 | 4 | 4 |
| **1.2** | **Panitumumab** | 2 | 2 | 3 | 3 | 4 | 3 | 4 | 5 | 4 | 4 |
| **1.2** | **Aflibercept** | 2 | 4 | 2 | 3 | 2 | 3 | 3 | 4 | 4 | 4 |
| **1.2** | **Regorafenib** | 1 | 3 | 4 | 3 | 5 | 4 | 3 | 2 | 2 | 4 |
| **1.3** | **Bevacizumab** | 2 | 4 | 4 | 3 | 4 | 5 | 4 | 4 | 4 | 2 |
| **1.3** | **Cetuximab** | 2 | 3 | 2 | 2 | 1 | 3 | 3 | 4 | 4 | 3 |
| **1.3** | **Panitumumab** | 2 | 4 | 3 | 3 | 3 | 4 | 3 | 4 | 4 | 3 |
| **1.3** | **Aflibercept** | 2 | 4 | 3 | 3 | 2 | 4 | 3 | 4 | 4 | 3 |
| **1.3** | **Regorafenib** | 3 | 5 | 4 | 4 | 5 | 4 | 2 | 2 | 3 | 4 |
| **2.1** | **Bevacizumab** | 4 | 3 | 3 | 3 | 4 | 3 | 4 | 4 | 3 | 3 |
| **2.1** | **Cetuximab** | 3 | 5 | 5 | 4 | 3 | 4 | 4 | 5 | 3 | 4 |
| **2.1** | **Panitumumab** | 4 | 4 | 4 | 4 | 2 | 3 | 4 | 4 | 3 | 3 |
| **2.1** | **Aflibercept** | 3 | 2 | 3 | 3 | 5 | 2 | 3 | 4 | 2 | 4 |
| **2.1** | **Regorafenib** | 2 | 3 | 2 | 1 | 1 | 3 | 1 | 4 | 1 | 2 |
| **2.2** | **Bevacizumab** | 5 | 5 | 4 | 5 | 5 | 5 | 5 | 5 | 5 | 5 |
| **2.2** | **Cetuximab** | 3 | 4 | 3 | 4 | 4 | 4 | 4 | 5 | 3 | 4 |
| **2.2** | **Panitumumab** | 2 | 4 | 3 | 2 | 3 | 1 | 1 | 5 | 3 | 3 |
| **2.2** | **Aflibercept** | 2 | 3 | 1 | 3 | 1 | 2 | 1 | 3 | 2 | 2 |
| **2.2** | **Regorafenib** | 3 | 3 | 1 | 1 | 2 | 3 | 3 | 2 | 2 | 2 |
| **2.3** | **Bevacizumab** | 5 | 5 | 5 | 5 | 5 | 5 | 5 | 5 | 5 | 5 |
| **2.3** | **Cetuximab** | 4 | 4 | 4 | 4 | 4 | 4 | 4 | 4 | 4 | 4 |
| **2.3** | **Panitumumab** | 3 | 4 | 4 | 2 | 3 | 1 | 4 | 4 | 2 | 1 |
| **2.3** | **Aflibercept** | 1 | 1 | 2 | 2 | 1 | 2 | 3 | 1 | 1 | 1 |
| **2.3** | **Regorafenib** | 2 | 1 | 2 | 3 | 2 | 3 | 3 | 2 | 3 | 1 |
| **3.1** | **Bevacizumab** | 4 | 4 | 5 | 1 | 3 | 4 | 3 | 2 | 4 | 1 |
| **3.1** | **Cetuximab** | 3 | 4 | 4 | 2 | 2 | 5 | 3 | 2 | 3 | 1 |
| **3.1** | **Panitumumab** | 2 | 3 | 5 | 4 | 1 | 4 | 3 | 2 | 3 | 2 |
| **3.1** | **Aflibercept** | 2 | 3 | 4 | 4 | 5 | 4 | 4 | 4 | 3 | 4 |
| **3.1** | **Regorafenib** | 2 | 5 | 3 | 5 | 4 | 5 | 4 | 4 | 2 | 5 |
| **3.2** | **Bevacizumab** | 5 | 5 | 4 | 1 | 5 | 4 | 2 | 5 | 4 | 4 |
| **3.2** | **Cetuximab** | 3 | 2 | 3 | 3 | 3 | 5 | 2 | 5 | 3 | 4 |
| **3.2** | **Panitumumab** | 2 | 3 | 3 | 3 | 2 | 3 | 2 | 3 | 3 | 2 |
| **3.2** | **Aflibercept** | 1 | 1 | 4 | 3 | 1 | 3 | 1 | 2 | 2 | 2 |
| **3.2** | **Regorafenib** | 3 | 5 | 5 | 4 | 4 | 4 | 4 | 4 | 1 | 2 |
| **3.3** | **Bevacizumab** | 4 | 4 | 5 | 5 | 5 | 5 | 5 | 5 | 5 | 5 |
| **3.3** | **Cetuximab** | 4 | 4 | 5 | 5 | 4 | 5 | 5 | 5 | 4 | 5 |
| **3.3** | **Panitumumab** | 4 | 4 | 3 | 3 | 3 | 3 | 4 | 4 | 4 | 3 |
| **3.3** | **Aflibercept** | 3 | 3 | 2 | 3 | 2 | 3 | 2 | 3 | 3 | 2 |
| **3.3** | **Regorafenib** | 4 | 4 | 3 | 2 | 1 | 4 | 5 | 4 | 3 | 3 |
